# A Power-Based Framework for Quantifying Parameter Uncertainties in Finite Vibroacoustic Metamaterial Plates

**DOI:** 10.3390/ma16145139

**Published:** 2023-07-21

**Authors:** Heiko Atzrodt, Arun Maniam, Marvin Droste, Sebastian Rieß, Moritz Hülsebrock

**Affiliations:** Fraunhofer Institute for Structural Durability and System Reliability LBF, 64289 Darmstadt, Germany

**Keywords:** active structural intensity, finite VAMM, power loss, stop band, uncertainty

## Abstract

Vibroacoustic metamaterials (VAMMs) are artificial materials that are specifically designed to control, direct, and manipulate sound waves by creating a frequency gap, known as the stop band, which blocks free wave propagation. In this paper, a new power-based approach that relies on the active structural intensity (STI) for predicting the stop band behavior of finite VAMM structures is presented. The proposed method quantifies the power loss in a locally resonant finite VAMM plate in terms of percentage, such as *STI*_99%_ and *STI*_90%_, for stop band prediction. This allows for the quantitative analysis of the vibration attenuation capabilities of a VAMM structure. This study is presented in the context of a two-dimensional VAMM plate with 25 resonators mounted in the middle section of the plate. It has been demonstrated that this method can predict the stop band limits of a finite VAMM plate more accurately than using negative effective mass, unit cell dispersion analysis, or the frequency response function methods. The proposed approach is then implemented to establish a framework for investigating the influence of parameter uncertainties on the stop band behavior of the VAMM plate. Based on the *STI*_99%_ method, which aims for significant vibration reduction, stricter tolerances in the mass fabrication process are required to ensure the robustness of VAMM. Conversely, the *STI*_90%_ method suggests that larger fabrication tolerances can be leveraged to achieve a broader stop band range while still meeting the desired performance level, leading to cost savings in manufacturing.

## 1. Introduction

Lightweight technology has emerged as an undisputed trend topic in recent years [1,2,3,4,5]. Due to the traditional mass law, lightweight materials and designs have a major drawback: they worsen vibrational and acoustic performance. Conventional solutions to suppress unwanted mechanical vibrations and noise often require heavy and bulky constructions, contradicting the recent trend toward the introduction of lightweight materials and designs [6,7].

The concept of vibroacoustic metamaterial based on the local resonance effect has shown a promising solution to achieve lightweight and good vibroacoustic performance [8,9,10]. The working principle of VAMM is mainly based on their microstructure, which consists of repeated elements called unit cells (UCs). Each UC is composed of a spring-mass-damper system called a local resonator. The resonators could either be attached to the host structure or integrated into the host structure. In the vicinity of the resonator’s resonance frequency, VAMM forms a so-called stop band. A stop band is a frequency range in which no free propagation of elastic waves is possible [11,12,13]. Only undamped systems have perfect stop bands. All real-world structures possess a certain degree of damping. In real damped systems, a stop band is, therefore, typically characterized as a frequency band where vibrations are significantly reduced. The interaction between the local resonators and the traveling wave leads to the formation of a stop band [14]. To achieve a stop band, the distances between the resonant elements must be smaller than half the wavelengths of the waves to be affected [13,15].

Since VAMMs usually have periodic configurations, wave propagation techniques such as Bloch waves, Floquet models, and transfer matrix methods are commonly used to derive the dispersion relations from the periodic symmetry of the metamaterial UCs to generate the wave dispersion curves [16,17,18,19]. With the help of dispersion curves, the stop bands of VAMMs could be predicted. Even though VAMMs do not require periodicity, they are often implemented for manufacturing reasons or modeling purposes [19,20,21]. The prediction of the stop band behavior with the methods mentioned earlier is purely based on the dynamic behavior of the UC model, representing infinite periodic structures, and not taking into consideration the real boundary conditions [19,22]. Additionally, the UC model may not take into account the design complexities or non-uniform variations in material properties or geometries within the periodic structure. Consequently, the responses of actual metamaterials often deviate from the predictions of the UC dispersion curves [19]. This deviation can result in the VAMM not performing as expected and can limit the effectiveness and applicability of the material. In addition, the Bloch approach becomes complex when damping is included, leading to complex frequencies and wavenumbers in the dispersion analysis [23,24]. It is, therefore, of enormous interest to researchers to investigate the dynamic behaviors of finite VAMMs using other approaches.

As the performance of VAMM depends primarily on the properties of the resonators, any uncertainty or variability in the resonator parameters, such as mass, spring stiffness, and the damping ratio, has an impact on the vibroacoustic performance of the VAMM [25,26]. In the context of industrial applications, the material and manufacturing tolerances can also introduce disturbances that affect the periodicity [27,28]. In [29,30,31], the influence of uncertainties in periodic structures has been investigated. In order to put the VAMM concept into real-world applications, it is important to quantify the impact of uncertainties in factors such as resonator parameters, the spatial distance between resonators, real-boundary conditions, and non-periodicity on the stop band of the finite VAMM. Studying the impact of these factors on the finite VAMM performance is crucial in identifying optimal design and manufacturing strategies of finite VAMM that can minimize the impact of parameter variability and ensure consistent performance.

**Figure 1:**
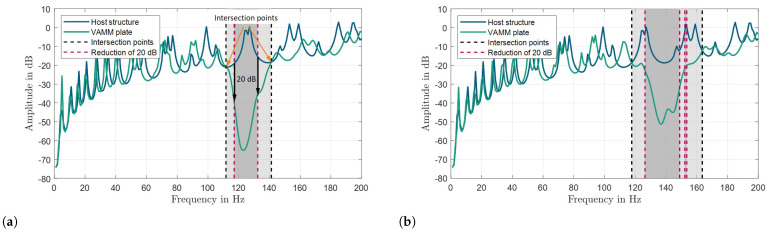
Example of FRF curves of the host structure and VAMM for (**a**) an ideal system and (**b**) a system with a resonator mass reduction of 21%. Two possible approaches, namely the intersection point method (black dashed lines) and the 20 dB reduction method (maroon dashed lines) are shown for predicting the stop band width. The gray region represents the stop band.

The stopband width of a metamaterial can serve as an important performance indicator, particularly when examining the influences of parameter uncertainty. Compared to other approaches mentioned above, the analysis of the frequency response function (FRF) emerges as the ideal method for evaluating uncertainties, as it presents fewer limitations compared to other techniques. However, predicting the width of the stop band solely based on the FRF analysis poses a significant challenge. One potential approach is to compare the FRFs of the VAMM with its host structure. By examining the intersection between these two curves in the vicinity of the resonance frequency, the upper and lower limits of the stop band can be defined (see Figure 1a). Another possible criterion for determining the stop band could be a specific reduction in decibels (dB) value (e.g., a 20 dB reduction) of the VAMM FRF in relation to that of the host structure (see Figure 1a).

However, identifying the start and end points of the stop band can be challenging due to the sensitivity of this method to the FRF curves. Even slight modifications of the resonator parameters can lead to significant variations in the width of the stop band. For instance, Figure 1b displays the FRFs of the host structure and the VAMM when the resonator’s mass is reduced by 21%. According to the theory, a decrease in the mass of the resonators should result in a smaller stop band. However, upon examining the intersection points in Figure 1b, it is observed that the predicted stop band using this method is wider, contradicting the theoretical expectation. Moreover, when considering a reduction of 20 dB, two separate stop bands can be identified instead of a single, smaller continuous stop band. Introducing uncertainties into the system further complicates the analysis. Therefore, a robust approach is necessary to overcome these limitations and accurately determine the boundaries of the stop band.

In the context of developing an automated stop band identification process for VAMMs with the presence of uncertainties, it is imperative to clearly outline the definition of the stop band. The automated determination of stop bands becomes vital for the optimization and evaluation of VAMM performance when considering parameter uncertainties. Therefore, this overall research aims to provide an effective and robust algorithm for accurately identifying the stop band boundaries, thus enabling automated processes for uncertainty assessment. One potentially valuable tool for investigating vibroacoustic problems is the utilization of active structural intensity (STI) [32].

Recently, the utilization of a power-based approach that relies on an active STI in the scope of VAMM has demonstrated promising results in evaluating the existence of a stop band. H. Al Ba’ba’a et al. presented a numerical investigation of vibrational power flow in one-dimensional (1D) metamaterials, demonstrating that the STI analysis can accurately predict power flow and stop band frequencies, providing an alternative to traditional wave dispersion analysis and negative effective mass methods, while also enabling an accurate depiction of the performance of dissipative metamaterials and facilitating the optimization of actual metamaterial designs [19]. In addition, an active STI can also be used to predict the occurrence of stop bands in 1D dissipative phononic structures with viscoelastic components, providing a clear distinction between Bragg scattering effects and wave attenuation due to material damping [24]. Most recently, the STI approach has been extended to a two-dimensional (2D) metamaterial plate, presenting a detailed assessment of the experimental measurements of the different STI representations and comparing the experimentally determined power flow pattern with the mechanical energy amplitude to find the metamaterial plate’s stop band, which was validated against the dispersion curve predictions [33].

Despite the limited number of published papers on the utilization of the STI approach in the field of VAMM, the above analyses have shed light on its application. These analyses include numerical investigations into the STI approach in 1D locally resonant metamaterials and 1D dissipative phononic structures, as well as experimental evaluations of the STI method in 2D locally resonant elastic metamaterials. However, as far as what is currently known, there has been no numerical study on the utilization of the STI method in 2D VAMM plates. Furthermore, the current state of research lacks a precise definition of the stop band limits for finite VAMM, which poses a challenge in conducting the uncertainty analysis and automating the detection of stop bands. This paper aims to address these gaps by providing a clear definition of the stop band in terms of a percentage, thereby facilitating the automation and accurate localization of stop bands in finite VAMM.

This work is mainly divided into two parts. The first part of this work utilizes the active STI to develop a new power-based approach that quantifies the power loss in a 2D VAMM plate using the finite element method. In this work, a clear definition of the stop band in terms of percentage has been established, which can be leveraged for a variety of applications based on the intended purpose and desired degree of vibration attenuation of the VAMM structures. In order to facilitate the discussion and validate the results, the stop band predicted using the power-based method is compared with the unit cell dispersion analysis method (UCDA), the negative effective mass method ($m_{\mathrm{eff}}$), and the FRF.

In the second part of this work, the developed power-based approach is then applied in uncertainty analysis to create a robust algorithm that accurately evaluates and determines the impact of parameter uncertainties on the stop band behavior of a finite VAMM plate. The current study concentrates solely on the mass parameter uncertainty to demonstrate the applicability and robustness of the developed algorithm for uncertainty analysis, with the intention of extending its use to examine the effects of other parameter uncertainties on stop band behavior.

## 2. Structural Intensity

STI describes the magnitude and direction of the power flow in a structure excited by an external force. STI is defined as the vibration power flow per unit area transmitted in an elastic structure subjected to a dynamic loading [33,34]. Similar to airborne sound intensity, STI is a vector resulting from the product of the stress tensor, *S*, and the velocity vector, $\vec{v}$, as shown in Equation (1) [35].

(1) $$\begin{array}{c} \;\;\;\vec{I}=-S\cdot \vec{v}=-\left[\begin{array}{ccc} \sigma_{xx} & \tau_{xy} & \tau_{xz}\\ \tau_{yx} & \sigma_{yy} & \tau_{yz}\\ \tau_{zx} & \tau_{zy} & \sigma_{zz} \end{array}\right]\cdot \left[\begin{array}{c} v_{x}\\ v_{y}\\ v_{z} \end{array}\right] \end{array}$$

In other words, STI is the work conducted by a unit area in the direction of velocity [36]. This means that STI is a vector quantity that indicates not only the flow of energy across a unit area over time but also the direction in which the energy is transported. The negative sign in Equation (1) comes from the fact that σ and τ are tensile stresses, which are distinguished from compressive forces by the sign [37].

The STI in Equation (1) shows energy transport as a function of time and is referred to as instantaneous STI [38]. This means that the STI varies depending on its location in the elastic medium and over time. However, when dealing with harmonic structure-borne sound waves, the structural intensity in the frequency domain, I(f), is of interest and it could be derived from time-averaged I(t) (e.g., over one period) [39]. The STI at a steady state can be calculated in the frequency domain using a Fourier transform in Equation (1) [33,40], which yields Equation (2), where $\underline{S}\left(f\right)$ is a frequency-dependent stress tensor and ${\underline{\vec{v}}}^{*}\left(f\right)$ is a complex conjugate of the frequency-dependent velocity.

(2) $$\begin{array}{c} {\vec{\underline{I}}}_{s}\left(f\right)=-\frac{1}{2}\cdot \underline{S}\left(f\right)\cdot {\underline{\vec{v}}}^{*}\left(f\right) \end{array}$$

The underline denotes complex quantities and the asterisk (*) conjugates complex quantities. The subscript letter, *s* in ${\vec{\underline{I}}}_{s}$, refers to steady-state STI. The factor 1/2 in Equation (2) is derived from the time-averaging process of the function. Thus, the STI is the product of the peak values of the stresses and the vibrational velocities. According to Equation (2), the complex STI is composed of a real component known as active STI, ${\vec{I}}_{S,\;a}$ (Equation (3)), and an imaginary component known as reactive STI or reactive power, ${\vec{I}}_{S,\;r}$ (Equation (4)) [39].

(3) I→S,a=ReI_→sf

(4) I→S,r=ImI_→sf

The active STI, ${\vec{I}}_{S,\;a}$, describes the energy flow from the source to the sink (moving wave) on a time-average basis [39]. On the other hand, the reactive STI, ${\vec{I}}_{S,\;r}$, describes the amount of energy that constantly oscillates in a structure (standing wave), allowing conclusions to be derived about the amplitude distribution of the natural mode of vibration. This presented work utilizes the active STI to quantify the vibrational energy flow and energy transmission pathways within a locally resonant finite VAMM structure.

## 3. STI Calculation in Thin Structures

For thin-walled structures, such as thin plates, it can be assumed that the energy transport across the plate thickness is negligible $\left(I_{z}=0\right)$ [41]. As discussed by Noiseux [42], the STI calculation can be simplified from 3D to 2D shell structures by performing integration over the thickness of the shell, thereby reducing it into *x* and *y* components only. The integrated intensity is now equal to the net energy flow per unit width (length) of the mid-surface of the shell with the unit of W/m, as shown by Equation (5) [43], where ${\underline{M}}_{x}$, ${\underline{M}}_{y}$, ${\underline{M}}_{xy}$, ${\underline{N}}_{xy}$, ${\underline{Q}}_{x}$, ${\underline{Q}}_{y}$, ${\underline{N}}_{x}$, and ${\underline{N}}_{y}$ denote bending moments, torsional moments, in-plane shear forces, out-of-plane shear forces, and in-plane axial forces per unit width of the plate, respectively. Also, ${\underline{v}}_{x}$, ${\underline{v}}_{y}$, ${\underline{v}}_{z}$, and ${\underline{\dot{\phi }}}_{x}$, ${\underline{\dot{\phi }}}_{y}$ represent translational velocities and rotational velocities. As a result, it is possible to specify the STI integrated over the shell thickness as a function of the internal forces and moments and, therefore, easily compute using FEM simulations [44].

(5) $$\begin{array}{c} {\vec{I}}_{s^{\prime }}=\left[\begin{array}{c} {\vec{I}}_{s^{\prime },\;x}\\ {\vec{I}}_{s^{\prime },y} \end{array}\right]=-\frac{1}{2}\cdot \left[\begin{array}{c} {\underline{N}}_{x}{\underline{v^{*}}}_{x}+{\underline{N}}_{xy}{\underline{v^{*}}}_{y}+{\underline{Q}}_{x}{\underline{v^{*}}}_{z}+{\underline{M}}_{x}{\underline{{\dot{\phi }}^{*}}}_{y}-{\underline{M}}_{xy}{\underline{{\dot{\phi }}^{*}}}_{x}\\ {\underline{N}}_{y}{\underline{v^{*}}}_{y}+{\underline{N}}_{xy}{\underline{v^{*}}}_{x}+{\underline{Q}}_{y}{\underline{v^{*}}}_{z}-{\underline{M}}_{y}{\underline{{\dot{\phi }}^{*}}}_{x}+{\underline{M}}_{xy}{\underline{{\dot{\phi }}^{*}}}_{y} \end{array}\right] \end{array}$$

**Figure 2:**
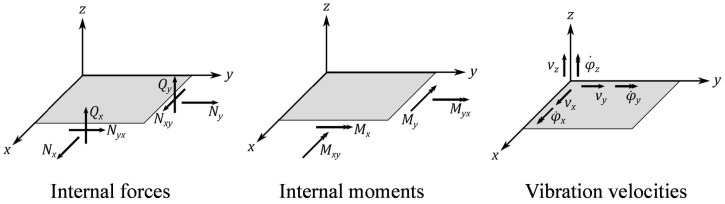
Definition of internal forces and moments as well as the rotational and translational vibrational velocities [45].

The first two summands in the equation describe the energy transport of the in-plane waves by the membrane forces ${\underline{N}}_{x}$, ${\underline{N}}_{y}$, and ${\underline{N}}_{xy}$. The other summands represent the energy transport of the out-of-plane waves caused by the shear forces (${\underline{Q}}_{x}$ and ${\underline{Q}}_{y}$), the bending moments (${\underline{M}}_{x}$ and ${\underline{M}}_{y}$), and the torsional moment (${\underline{M}}_{xy}$). These so-called in-plane waves consist of transverse and extensional waves. The quantities from Equation (5) are defined positively according to Figure 2.

## 4. Energy Balance in Elastic Medium

STI in an elastic medium can be described using the first law of thermodynamics [41]. The law of conservation of energy is represented by Equation (6) [46]. The law states that the total energy of a closed system is constant, implying that the sum of all energy changes in a closed system is zero.

(6) $$\begin{array}{c} {\;\int \int \int \;}_{V}\frac{de}{dt}\;dV={\;\int \int \;}_{A}-S\frac{d\vec{u}}{dt}\vec{n}\;dA+{\;\int \int \int \;}_{V}\left(\pi_{\mathrm{in}}-\pi_{\mathrm{diss}}\right)\;dV \end{array}$$

where *e* is the energy density inside the control volume, $\vec{u}$ is the displacement vector of any particle at the boundary of the control volume, $\pi_{\mathrm{in}}$ is the input power density (or the energy supplied per unit volume per unit time), $\pi_{\mathrm{diss}}$ is the dissipated power density (or the energy dissipated per unit volume per unit time), $\vec{n}$ is the vector perpendicular (normal) to the surface of the control volume for a given point on the surface, and *S* is the stress tensor.

Although the law was developed for thermodynamic processes, it can be used to represent the energy balance in an elastic medium using a control volume approach. The rate of change of total energy within the surface enclosing the volume is equivalent to the energy flow through a closed surface [46]. The first summand on the left side of Equation (6) describes the change in energy density within the control volume. This change in energy density is due to the work conducted by the stress on the medium. In terms of stress and velocity, the local outflow of the energy density from the control volume is known as STI, as described in Equation (7) [46].

(7) $$\begin{array}{c} {\vec{I}}_{S}=-S\;\frac{d\vec{u}}{dt} \end{array}$$

By substituting Equation (7) into Equation (6), the equation can be rewritten as

(8) $$\begin{array}{c} \frac{dE}{dt}=-{\;\int \int \;}_{A}{\vec{I}}_{S}\;\vec{n}\;dA+\;P_{\mathrm{in}}-P_{\mathrm{diss}} \end{array}$$

where $P_{\mathrm{in}}$ is the input power (in-flow of the energy into the control volume) and $P_{\mathrm{diss}}$ is the dissipated power density (out-flow of the energy from the control volume). Equation (8) represents the energy relationship for all elastic media and is valid for steady-state or transient analysis. When dealing with harmonic structure-borne sound waves, the change in energy over time, dE/dt, is zero for steady-state (time-averaged value) vibrational energy propagation and, hence, Equation (8) becomes

(9) $$\begin{array}{c} {\;\int \int \;}_{A}\langle {\vec{I}}_{S}\rangle \;\vec{n}\;dA=\langle P_{\mathrm{in}}-P_{\mathrm{diss}}\rangle \end{array}$$

where the angled brackets 〈〉 reflect the time-averaging. The correlation between time-averaged STI and the consideration of STI in the frequency domain is as follows:

(10) $$\begin{array}{c} \langle {\vec{I}}_{S}\left(t\right)\rangle =\int_{f}{\vec{I}}_{S^{\prime },a}\left(f\right)\;df \end{array}$$

## 5. Derivation of Dissipated and Injected Power

**Figure 3:**
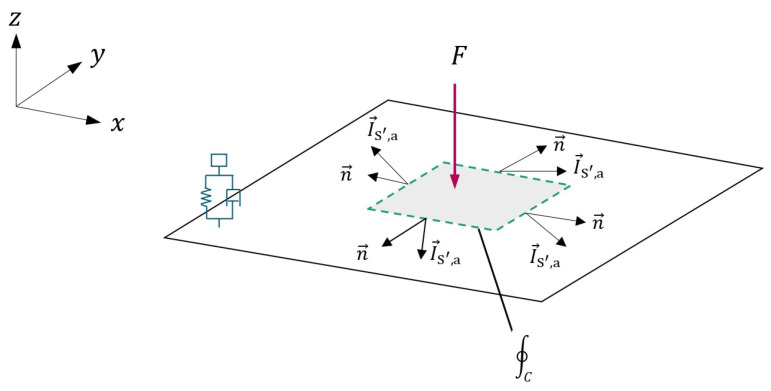
Illustration of Equation (11).

As this work is only concerned with thin plates, the STI in Equation (9) can be simplified to a contour integral as depicted in Figure 3 and as described by Equation (11) [47].

(11) $$\begin{array}{c} \oint_{C}{\vec{I}}_{S^{\prime },a}\;\vec{n}\;dl=\;P_{\mathrm{in}}-P_{\mathrm{diss}} \end{array}$$

where *C* describes the contour of the shell element and *l* represents the length of each element along the contour line. In the context of shell structure, the surface integral in Equation (9) can be simplified to a contour integral along the closed area, as depicted in Figure 3.

If the contour integral includes a region with no energy source but only an energy sink, the dissipation power (negative value) can be determined using Equation (11). Similarly, if the contour integral covers a region with no energy sink but only an energy source, the injected power (positive value) can be obtained.

## 6. VAMM Plate

**Figure 4:**
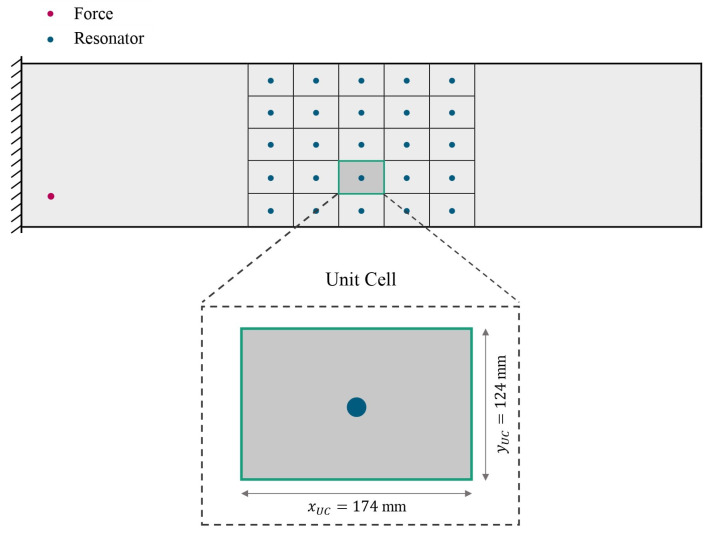
Schematic representation of the VAMM plate and its UC with its dimensions.

In this work, a rectangular Kirchhoff–Love plate model made of steel is used as the host structure. The plate has dimensions of 2610 mm × 620 mm × 5 mm for length, $l_{x}$ width, $l_{y}$ and thickness, *t*. A unit point force F is applied in the z-direction at the position $x_{F}=$ 100 mm and $y_{F}=100$ mm from the bottom-left corner of the plate (see Figure 4). The left edge of the plate is clamped, meaning that both translational and rotational displacements at this edge are set to zero. The parameters of the resonator are chosen to create a stop band in the frequency range of around 130 Hz. An additional mass of 7% and Lehr’s damping ratio of 1% are chosen to parameterize the resonators. The 5 × 5 resonators are attached to the middle section of the plate. Table 1 summarizes the material parameters used.

## 7. Stop Band Prediction

This section presents the different methods applied for predicting the stop bands of an infinite periodic VAMM plate and a finite VAMM plate in both undamped and damped systems.

### 7.1. Negative Effective Mass, m_*eff*_ Method

A two DOF-system based on work by Sun [48] can be examined to demonstrate the negative effective mass of a VAMM plate. The $m_{\mathrm{eff}}$ method used here is only valid for undamped systems. The effective mass of a single undamped UC can be determined using Equation (12)

(12) $$\begin{array}{c} m_{\mathrm{eff}}\;=m_{1}+\frac{m_{2}\cdot {\omega_{2}}^{2}}{{\omega_{2}}^{2}-\Omega^{2}}=m_{1}+\frac{m_{2}}{1-\frac{\Omega^{2}}{{\omega_{2}}^{2}}} \end{array}$$

where $m_{1}$ is the mass of the UC plate, $m_{2}$ is the mass of the resonator, $\omega_{2}$ is the resonance frequency of the resonator and Ω is the excitation frequency. Equation (13) represents the theoretical stop band range. This indicates that the starting frequency is directly influenced by the natural frequency of the second mass, $\omega_{2}$, while the width of this frequency range is dependent on the ratio of masses, $m_{2}/m_{1}$.

(13) $$\begin{array}{c} \omega_{2}<\Omega <\omega_{2}\cdot \sqrt{\frac{m_{2}}{m_{1}}+1}\; \end{array}$$

**Figure 5:**
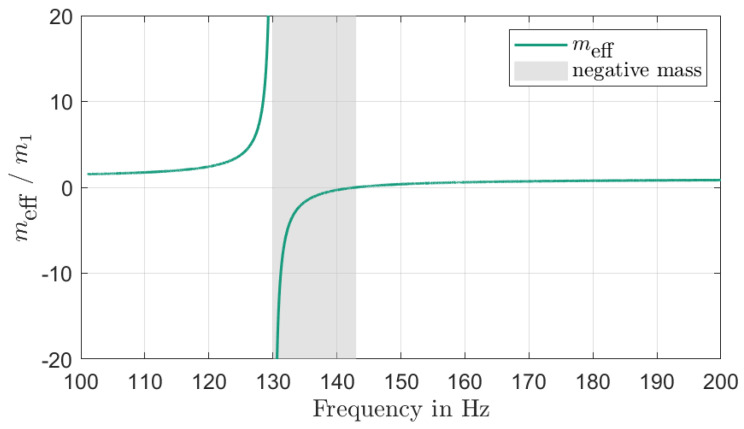
Effective mass of a single UC shown in Figure 4.

Figure 5 shows the effective mass, $m_{\mathrm{eff}}$, normalized to the mass of the UC plate, $m_{1}$, and plotted against the excitation frequency, Ω. When the normalized mass drops below zero, there is a stop band present in the frequency range of 130 Hz to 143 Hz.

### 7.2. Unit Cell Dispersion Analysis, UCDA Method

UCDA is often used to investigate the stop band behavior of the infinite periodic VAMM structure. This is due to the fact that the response of the UC characterizes the VAMM response [13]. The application of Bloch’s theorem allows the modeling of such structures by considering a UC [16]. The equation of motion of the UC illustrated in Figure 4 for the steady-state harmonic response is as follows:

(14) $$\begin{array}{c} \left[-\omega^{2}M+i\omega D+K\right]\;q=F \end{array}$$

**Figure 6:**
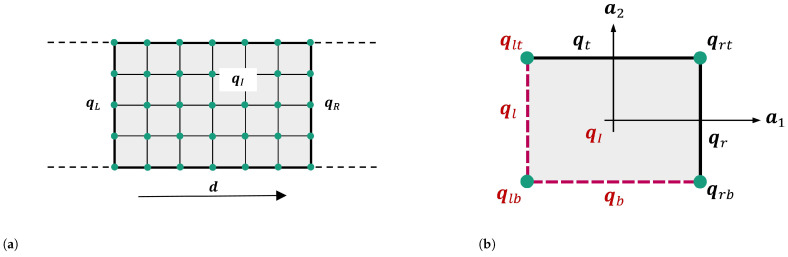
(**a**) FE mesh of a 1D periodic system based on [49]. (**b**) A UC of a rectangular lattice exhibiting periodicity along the directions, $a_{i}$. The master DOFs are marked in maroon. The indices describe the positions, such as left, right, top, and bottom.

Bloch’s theorem [16] states that the DOFs at the opposite edges or corners of the UCs are coupled based on wave propagation characteristics. This coupling of the UC’s opposite edges can be studied using a 1D periodic system as an example. Figure 6a and Equation (15) [16] depicts this relationship, where $q_{L}$ and $q_{R}$ denote the DOFs at the UC’s left edge and right edge in the direction of wave propagation, d, respectively. $\mu_{R}$ and $\mu_{I}$ in Equation (15) denote the attenuation factor and phase change, respectively, and Λ describes the complex eigenvalue, $e^{\mu_{R}+i\mu_{I}}$. For instance, if a wave with a propagation vector, μ, goes through a UC in the direction of d, it experiences an amplitude gain of $e^{\mu_{R}}$ and a phase change of $e^{\mu_{I}}$. The complex eigenvalue $\Lambda =e^{\mu_{R}+i\mu_{I}}$ is introduced here for simplification and better understanding.

(15) $$\begin{array}{c} q_{R}=e^{\mu_{R}+i\mu_{I}}\;q_{L}=\Lambda \;q_{L} \end{array}$$

With the help of this relationship, the vector of the reduced DOF of a UC, $q^{\left(red\right)}$ can be derived. Hence, using Langley’s reduction matrix $R\left(\Lambda \right)$ [50], the vector of nodal DOF, ***q*** can be characterized by Equation (16). Matrix $R\left(\Lambda \right)$ is a function of propagation vector μ [13]. This matrix relates the independent and reduced number of master DOFs of a UC, $q^{\left(red\right)}$ to the whole set of DOFs, q.

(16) $$\begin{array}{c} q=R\left(\Lambda \right)q^{\left(red\right)} \end{array}$$

In a 1D periodic system, Equation (16) can be written using unit matrix I, as follows:

(17) $$\begin{array}{c} q=\left[\begin{array}{c} q_{L}\\ q_{I}\\ q_{R} \end{array}\right]=\left[\begin{array}{cc} I & 0\\ 0 & I\\ \Lambda I & 0 \end{array}\right]\left[\begin{array}{c} q_{L}\\ q_{R} \end{array}\right]=\;Rq^{\left(red\right)} \end{array}$$

This matrix R can be utilized to rewrite the dynamic Equation (14) of a UC in reduced form, where the asterisk (*) denotes the complex conjugate and *T* denotes transposition.

(18) $$\begin{array}{c} R^{*T}\;\left[-\omega^{2}M+i\omega D+K\right]\;Rq^{\left(red\right)}=R^{*T}\;F \end{array}$$

It was demonstrated by Langley that the right-hand side of Equation (17) $\left(R^{*T}\;F\right)$ is equal to zero under no external forces acting on the internal DOFs of the UC. This leads to the reduction of Equation (14) into an eigenvalue problem, as follows:

(19) $$\begin{array}{c} \left[-\omega^{2}M^{\left(red\right)}+i\omega D^{\left(red\right)}+K^{\left(red\right)}\right]\;q^{\left(red\right)}=0 \end{array}$$

where the reduced mass matrix $M^{\left(red\right)}=R^{*T}\;M\;R$, reduced damping matrix $D^{\left(red\right)}=R^{*T}\;D\;R$, and reduced stiffness matrix $K^{\left(red\right)}=R^{*T}\;K\;R$. In order to implement this approach in the context of 2D periodicity (see Figure 6b), modifications should be conducted to ${q,\;q}^{\left(red\right)}$ and R as follows:

(20) $$\begin{array}{c} q=\left[\begin{array}{c} q_{I}\\ q_{l}\\ q_{lb}\\ q_{b}\\ q_{rb}\\ q_{r}\\ q_{rt}\\ q_{t}\\ q_{lt} \end{array}\right]\;=\left[\begin{array}{cccc} I & 0 & 0 & 0\\ 0 & I & 0 & 0\\ 0 & 0 & I & 0\\ 0 & 0 & 0 & I\\ 0 & 0 & Ie^{\mu_{1}} & 0\\ 0 & Ie^{\mu_{1}} & 0 & 0\\ 0 & 0 & Ie^{\mu_{1}+\mu_{2}} & 0\\ 0 & 0 & 0 & Ie^{\mu_{2}}\\ 0 & 0 & Ie^{\mu_{2}} & 0 \end{array}\right]\left[\begin{array}{c} q_{I}\\ q_{l}\\ q_{lb}\\ q_{b} \end{array}\right]=Rq^{\left(red\right)}\; \end{array}$$

There are two ways to solve this eigenvalue problem, namely, a direct approach, $UCDA_{\mathrm{dir}}$, and inverse approach, ${UCDA}_{\mathrm{inv}}$ [13]. The ${UCDA}_{\mathrm{inv}}$ is frequently used to investigate the wave propagation in undamped 2D structures. On the other hand, the ${UCDA}_{\mathrm{dir}}$ method is typically utilized for damped systems, but only for wave propagation in a single specific direction.

#### 7.2.1. Inverse Approach, *UCDA*_inv_

**Figure 7:**
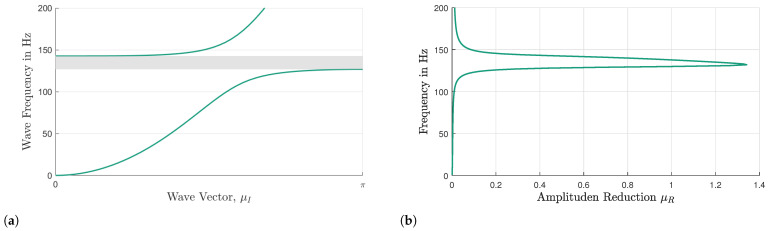
(**a**) Dispersion relation for wave propagation in the x-direction (0 – π) using the ${UCDA}_{\mathrm{inv}}$ method for the undamped infinite VAMM plate and (**b**) dispersion relation for wave propagation in the x-direction using ${UCDA}_{\mathrm{dir}}$ for the damped system. The gray region represents the stop band.

The ${UCDA}_{\mathrm{inv}}$ method is used to solve the complex propagation vector, μ, of the undamped infinite VAMM plate. In the eigenvalue problem, the system’s damping matrix, $D^{\left(red\right)}$, is equal to zero. Thus, the real part of the complex propagation vector, μ, denoted by $\mu_{R}$, which describes the amplitude reduction of the wave propagating through a single UC, is equal to zero, transforming the propagation vector into a purely imaginary entity, $\mu =i\mu_{I}$. Frequency ranges with no solutions for ω correspond to the stop band range of the undamped VAMM. In Figure 7a, it can be seen that from 126.7 Hz to 142.6 Hz, there is no solution available for ω, indicating that the formation of the stop band in the vicinity of the resonator’s resonance frequency is 130 Hz.

#### 7.2.2. Direct Approach, *UCDA*_dir_

Figure 7b shows the dispersion relation for a damped system. The stop band of a damped system is more difficult to determine than that of an undamped system. Based on Figure 7b, it is clear that only strongly damped solutions exist near the resonance frequency. Therefore, the magnitude of wave attenuation can be utilized to define the stop bands of damped systems.

The relationship between the amplitude of the wave entering a UC, ($A_{\mathrm{in}}$) and the amplitude of the wave leaving the UC ($A_{\mathrm{out}}$) can be expressed as

(21) $$\begin{array}{c} A_{\mathrm{out}}=A_{\mathrm{in}}\cdot \;e^{-\mu_{R}} \end{array}$$

In order to find the amplitude reduction, the $A_{\mathrm{red}}$ in a single UC in the percentage, Equation (21) can be rewritten as follows:

(22) $$\begin{array}{c} A_{\mathrm{red}}=\frac{A_{\mathrm{out}}}{A_{\mathrm{in}}}=e^{-\mu_{R}}\cdot 100 \end{array}$$

**Figure 8:**
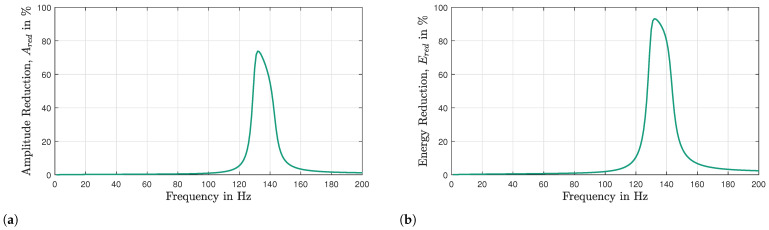
(**a**) Amplitude reduction, $A_{\mathrm{red}}$, and (**b**) energy reduction, $E_{\mathrm{red}}$, of the wave propagating through a single-damped UC.

The amplitude reduction, $A_{\mathrm{red}}$, in the percentage, is expressed as the amplitude ratio of the exiting wave, $A_{\mathrm{out}}$, to the entering wave, $A_{\mathrm{in}}$. In this context, $A_{\mathrm{red}}$ becomes zero percent if the amplitude of the entering wave, $A_{\mathrm{in}}$, is not attenuated at all, while $A_{\mathrm{red}}$ becomes 100% when the amplitude of the entering wave, $A_{\mathrm{in}}$, is completely attenuated. Figure 8a presents the amplitude reduction, $A_{\mathrm{red}}$, in a single-damped UC. To quantify the energy loss in a single UC, the relationship between the amplitude and energy of a wave can be utilized.

The amount of energy transported by a wave is linked to the amplitude of the wave, $E=C\cdot A^{2}$, where *C* is the coefficient proportionality. This shows that the energy of a wave is proportional to its amplitude squared, $E\propto A^{2}$. An assumption is made that the energy transported by a wave is equal to the square of the amplitude of that wave, $\;E=A^{2}$, where C=1. The accuracy of this assumption will be evaluated later by comparing the stop band predicted using this method with other approaches for the damped system. By utilizing the amplitude–energy relationship, the energy reduction, $E_{\mathrm{red}}$, of the wave propagating through a single-damped UC is computed and illustrated in Figure 8b.

**Figure 9:**
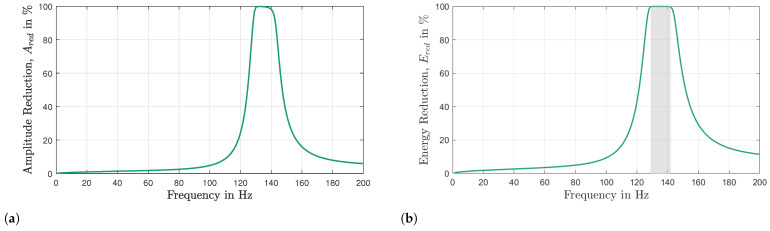
(**a**) Amplitude reduction, $A_{\mathrm{red}}$, and (**b**) energy reduction, $E_{\mathrm{red}}$, of the wave propagating through five damped UCs. The gray region represents the stop band.

In this work, a simplified calculation method is employed to quantify the amplitude and energy reduction of waves that occur when the waves pass through all the resonators in the x-direction. Despite the fact that there are 25 resonators in the middle section of the plate, the amplitude reduction caused by five UCs is computed because the ${UCDA}_{\mathrm{dir}}$ is only performed for one direction (in this case, the *x*-direction). This means that a wave traveling from left to right across the plate will only pass through five resonators. Table 2 provides a concise brief overview of the ${UCDA}_{\mathrm{dir}}$ for a single UC and five UCs. In Table 2, *n* is the number of UCs that the wave propagates through. Figure 9a depicts the amplitude reduction curve for five UCs and Figure 9b reveals the presence of a stop band between 127.2 Hz and 142.3 Hz by utilizing the $E_{\mathrm{red}}$ value of 99%.

### 7.3. Frequency Response Function, FRF

**Figure 10:**
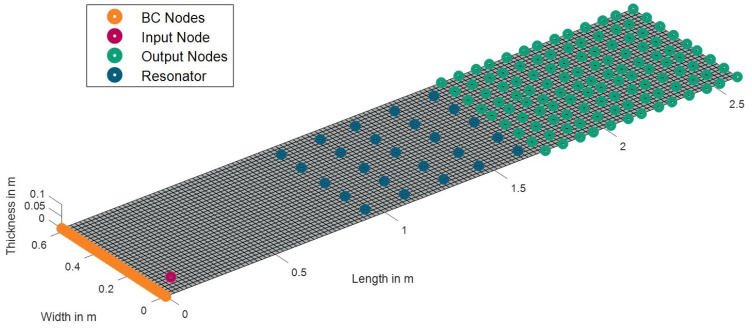
Illustration of the VAMM plate model with boundary condition (BC) nodes, input nodes, output nodes, and resonators.

**Figure 11:**
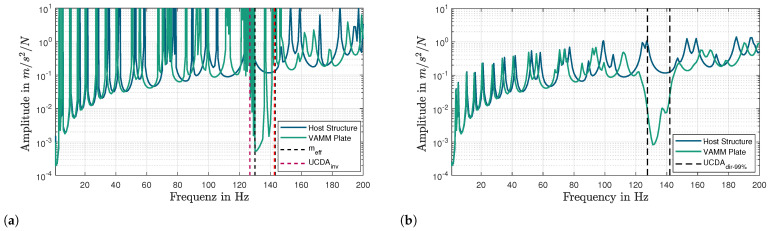
Comparison of the host structure’s FRF with the finite VAMM plate using different stop band prediction methods. (**a**) Undamped case: $m_{\mathrm{eff}}$ and ${UCDA}_{\mathrm{inv}}$. (**b**) Damped case: ${UCDA}_{\mathrm{dir}}$.

The FRF is the ratio of the output response of a structure to the applied input force. The FRF method is commonly used to analyze the stop band of a finite VAMM structure. As shown in Figure 10, the response of the VAMM plate at the nodes between the last column of the resonators and the right edge of the plate is measured and averaged. Typically, the stop band in an FRF diagram is represented by a sudden drop in the FRF curve. Figure 11a,b show the FRFs of an undamped and a damped VAMM plate. The FRF of an undamped system is compared with $m_{\mathrm{eff}}$ and ${UCDA}_{\mathrm{inv}}$ while the FRF response of a damped system is compared with ${UCDA}_{\mathrm{dir}-99\%}$. These comparisons are made to verify whether the stop band predicted using different methods corresponds well to the FRF of the VAMM plate. It is to be noted that the upper limits on the y-axis of the figures are intentionally chosen to highlight the frequency range relevant to the analysis.

## 8. Power-Based Method

**Figure 12:**
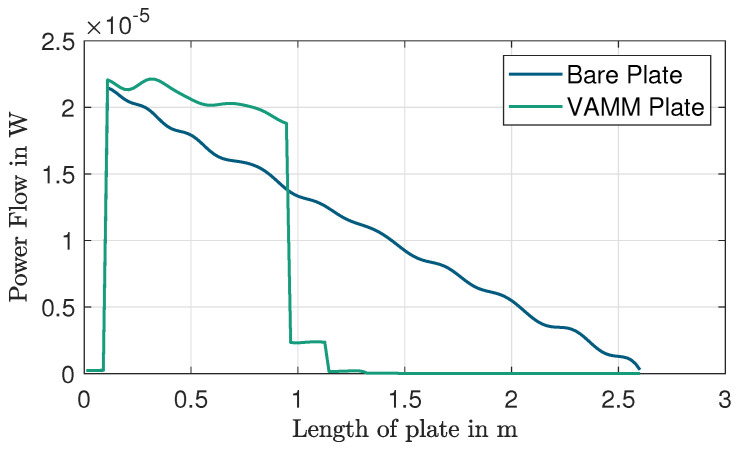
Power flow on the bare plate and VAMM plate along the length of the plate for a frequency of 131 Hz.

**Figure 13:**
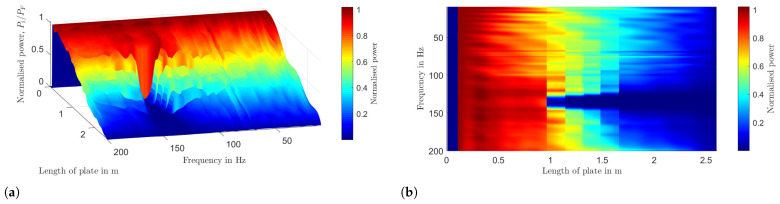
(**a**) Illustration of the VAMM plate with a contoured integral line and normal vectors defined for the calculation of the power loss and (**b**) the power loss of the damped VAMM plate.

As this work primarily focuses on waves propagating in the x-direction only, the power flow can be summed across the width of the plate, transforming the 2D plate model into a 1D beam-like model, revealing a similar power flow pattern to a 1D system, like a beam. Figure 12 shows the power flow along the length of the plate for a frequency of 131 Hz, which is within the desired stop band frequency range. The vertical spike in power corresponds to the input power contributed by harmonic force excitation, $P_{F}$, and the decline of power across the length of the bare plate is due to the material loss factor of the plate. The power flow of the VAMM plate in Figure 12 also illustrates a step-like decrease in power, which suggests that the energy is being dissipated at every column of resonators. It could be seen that in the region before the resonators and after the force excitation point (between 0.1 m and 0.9 m), the power in the VAMM plate is higher than the bare plate. This accumulation of power could be understood as a compensatory effect. Such an increase compensates for the local resonance band gap effect, which blocks the transmission of energy beyond the resonator region [33]. Figure 13a,b denote the graphical representations of power flow along the length of the VAMM plate, calculated for frequencies ranging from 0 to 200 Hz. The power, $P_{i}$, is normalized to the injected force, $P_{F}$. In Figure 13b, the horizontal dark blue region starting at 0.9 m indicates that the wave is effectively blocked as the energy is absorbed by the resonators. The frequency range of this horizontal dark blue region is consistent with the theoretical stop band and the drop in the FRF graph.

**Figure 14:**
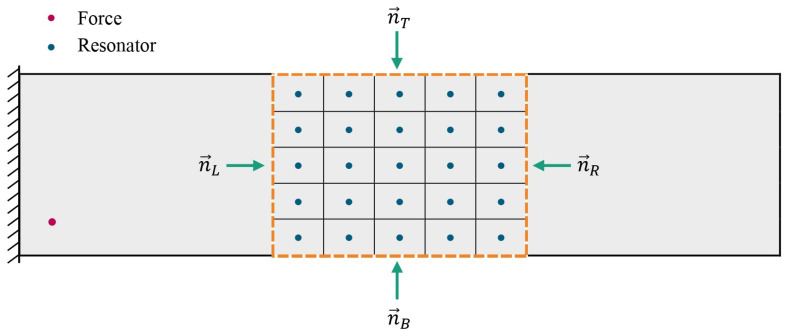
Illustration of the VAMM plate with the contoured integral line and normal vectors defined for the calculation of power loss.

The power loss in the resonator region can be determined by using Equation (11) by defining the contour line around the region with resonators, as illustrated in Figure 14. The vectors normal to the closed area (top, bottom, left, and right) are represented by ${\vec{n}}_{T}$, ${\vec{n}}_{B}$, ${\vec{n}}_{L}$, and ${\vec{n}}_{R}$, respectively. Since no power can flow beyond the clamped and free edges [41,51], the injected power may only flow over the left or right vertical lines.

Therefore, Equation (11) can be rewritten as follows:

(23) $$\begin{array}{c} \underset{P_{\mathrm{loss},\mathrm{VAMM}}}{\underset{︸}{\oint_{C}{\vec{I}}_{S^{\prime },a}\;\vec{n}\;dl}}=\underset{P_{\mathrm{in},\mathrm{VAMM}}}{\underset{︸}{\int_{0}^{l_{y}}{\vec{I}}_{S^{\prime },a}\left(x_{R},y\right){\;\vec{n}}_{R}dy}}+\underset{P_{\mathrm{out},\mathrm{VAMM}}}{\underset{︸}{\int_{0}^{l_{y}}{\vec{I}}_{S^{\prime },a}\left(x_{L},y\right){\;\vec{n}}_{L}dy}} \end{array}$$

where $P_{\mathrm{in},\mathrm{VAMM}}$, and $P_{\mathrm{out},\mathrm{VAMM}}$ denote the power flow into and out of the resonator region, respectively. Therefore, by substituting the values of ${\vec{n}}_{L}$ and ${\vec{n}}_{R}$ into Equation (23), the power loss basically equals the difference between power flowing into and out of the resonator region, ($P_{\mathrm{loss},\mathrm{VAMM}}$ = $P_{\mathrm{in},\mathrm{VAMM}}$−$P_{\mathrm{out},\mathrm{VAMM}}$). To express the power loss in terms of percentage, the power inflow and outflow, $P_{\mathrm{in},\mathrm{VAMM}}$ and $P_{\mathrm{out},\mathrm{VAMM}}$, are normalized with respect to the power entering the resonator zone, $P_{\mathrm{in},\mathrm{VAMM}}$.

In order to quantify power loss in the VAMM plate, relative to the host structure, the power loss calculation is repeated for the host structure (i.e., bare plate) in a similar manner, and then the power flows in the VAMM plate are normalized with respect to the power flows in the host structure, $P_{\mathrm{in},\mathrm{Host}}$ and $P_{\mathrm{out},\mathrm{Host}}$, respectively (Equation (24)), to yield the relative power loss caused by the resonators.

(24) $$\begin{array}{c} P_{\mathrm{loss},\mathrm{Rel}}=\;\left(\frac{\frac{P_{\mathrm{in},\mathrm{VAMM}}}{P_{\mathrm{in},\mathrm{VAMM}}}}{\frac{P_{\mathrm{in},\mathrm{Host}}}{P_{\mathrm{in},\mathrm{Host}}}}-\frac{\frac{P_{\mathrm{out},\mathrm{VAMM}}}{P_{\mathrm{in},\mathrm{VAMM}}}}{\frac{P_{\mathrm{out},\mathrm{Host}}}{P_{\mathrm{in},\mathrm{Host}}}}\right)\cdot 100 \end{array}$$

**Figure 15:**
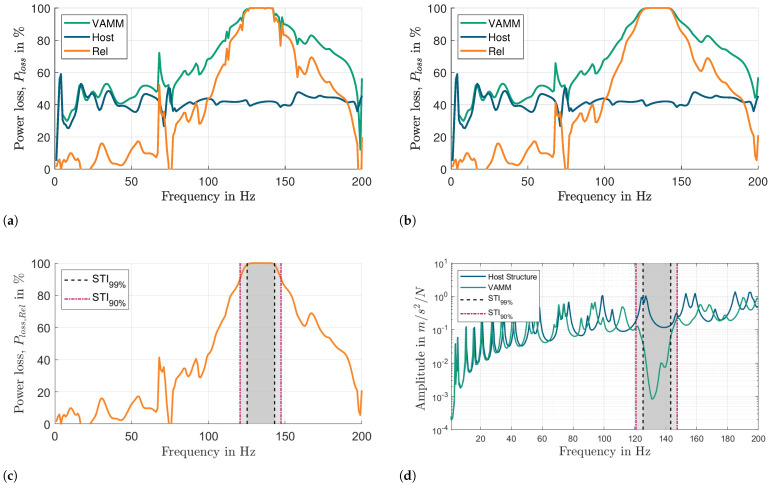
Comparison of the power loss in (**a**) undamped and (**b**) damped VAMM plates. Comparison of stop bands predicted by ${STI}_{99\%}$ and ${STI}_{90\%}$ with (**c**) $P_{\mathrm{loss},\mathrm{Rel}}$, and (**d**) with FRFs. The gray region represents the stop band.

The STI method only yields meaningful results in the presence of damping components, such as internal material damping or dissipative resonators. In the absence of any damping, the active STI, ${\vec{I}}_{S^{\prime },a}$, becomes zero. For validation purposes, a so-called undamped system is achieved by setting the material damping of the VAMM plate and Lehr’s damping ratio of the resonator to very low values instead of zeros ($10^{-4}$). Figure 15a,b shows the power loss in the VAMM plate, $P_{\mathrm{loss},\mathrm{VAMM}}$ (green line), the host structure, $P_{\mathrm{loss},\mathrm{Host}}$ (blue line), and the relative power loss, $P_{\mathrm{loss},\mathrm{Rel}}$ (orange line) for undamped and damped VAMM plates. It could be seen that approximately 40% of the power (blue line) is dissipated within the resonator region of the host plate due to material damping alone. Therefore, the normalization process is important to yield the relative power loss, $P_{\mathrm{loss},\mathrm{Rel}}$, induced by the resonators only.

Quantitative analysis of the vibration attenuation capabilities of a VAMM structure requires establishing a clear definition of the stop band in percentage terms. This step is crucial to accurately evaluate the structure’s vibroacoustic performance. The percentage value denotes the magnitude of the vibration attenuation achieved. Two threshold percentages were chosen in this work, namely, 90 and 99%, as shown in Figure 15c. The stop bands predicted based on these threshold values are compared with the FRF graphs, as illustrated in Figure 15d. The stop band gaps align well with the dip in the FRF of the VAMM plate. A relative power loss of 99% indicates that the resonators in VAMM structures absorbed 99% of the vibrational energy relative to the host plate. This definition of the stop band can be applied to various applications, depending on the intended purpose and the desired degree of vibration attenuation for the VAMM structures.

## 9. Comparison and Evaluation of Different Stop Band Prediction Methods

In this chapter, the stop bands predicted by the ${STI}_{99\%}$ and ${STI}_{90\%}$ methods for undamped and damped systems are compared to those predicted by conventional methods. Table 3 shows the conditions fulfilled by each prediction method. The 99% threshold achieves greater attenuation than the 90% threshold

### 9.1. Undamped VAMM Plate

**Figure 16:**
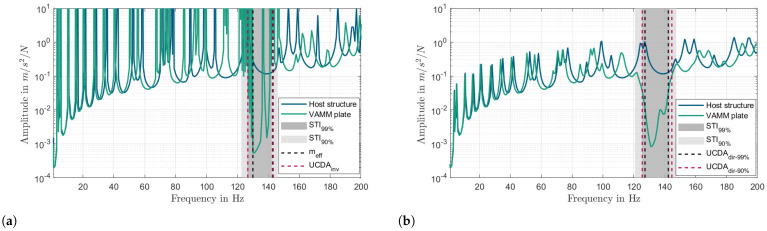
Comparison of the host structure’s FRF with the finite VAMM plate using different stop band prediction methods. (**a**) Undamped case: ${STI}_{99\%}$, $m_{\mathrm{eff}}$, and ${UCDA}_{\mathrm{inv}}$. (**b**) Damped case: ${STI}_{99\%}$, ${STI}_{90\%}$, $UCDA_{\mathrm{dir}-99\%}$, and $UCDA_{\mathrm{dir}-90\%}$.

Figure 16a shows the comparison of FRF curves of the host structure and VAMM plate with $m_{\mathrm{eff}}$, ${UCDA}_{\mathrm{inv}}$, ${STI}_{99\%}$, and ${STI}_{90\%}$. The FRF curve of the host structure is used to verify the presence of stop bands predicted by these methods. Table 4 presents a summary of the information depicted in Figure 16. The table provides information on how the stop bands are defined, as well as the range of frequencies where the stop bands begin and end for each method. As can be seen from Table 4, the stop bands predicted by ${UCDA}_{\mathrm{inv}}$ and ${STI}_{99\%}$ show good agreement but the stop band predicted by ${STI}_{90\%}$ is slightly wider than the former. This shows that the ${STI}_{99\%}$ method is more suitable in predicting the stop band with the same degree of attenuation as ${UCDA}_{\mathrm{inv}}$ than the ${STI}_{90\%}$ method. Moreover, it can be seen that the stop band predicted using the $m_{\mathrm{eff}}$ method starts exactly at the resonance frequency (130 Hz) as denoted by Equation (12). Due to the dynamic behavior of the host structure, the starting frequency of ${UCDA}_{\mathrm{inv}}$ is lower than that of $m_{\mathrm{eff}}$. Since ${UCDA}_{\mathrm{inv}}$ provides a more realistic representation of the system compared to $m_{\mathrm{eff}}$, the stop band predicted by the former is more accurate than the latter.

### 9.2. Damped VAMM Plate

Figure 16b illustrates the FRF plot of two damped systems: the host structure and the VAMM plate. The plot additionally presents the stop bands predicted by various methods, namely ${UCDA}_{\mathrm{dir}-99\%}$, ${UCDA}_{\mathrm{dir}-90\%}$, ${STI}_{99\%}$, and ${STI}_{90\%}$. The stop bands predicted by these methods agree well with the drop in the FRF curve of the damped VAMM plate.

According to the comparison presented in Table 4, the estimated range of the stop band using ${STI}_{99\%}$ is 18.5% wider than that obtained with ${UCDA}_{\mathrm{dir}-99\%}$. Similarly, ${STI}_{90\%}$ predicts a stopband width that is 39% wider than ${UCDA}_{\mathrm{dir}-90\%}$. These results illustrate a consistent trend where the stop band predicted by the STI method tends to be wider compared to the prediction made by ${UCDA}_{\mathrm{dir}}$, which is solely based on the UC model. The wider stop band captured by the STI method suggests that the actual boundary conditions of the finite VAMM plate have an impact on the width of the stop band. As the ${UCDA}_{\mathrm{dir}}$ method assumes a periodic boundary condition and an infinite plate, the stop band predicted by this method deviates to some extent from the actual stop band of the finite VAMM plate.

In this study, the parameter uncertainty analysis is conducted using a power-based approach that relies on the STI. This approach is chosen to investigate the performance of the actual VAMM plate as it is crucial to capture the dynamic behavior of the entire VAMM plate, as opposed to solely considering the UC model. The UC model assumes infinite length and periodicity and overlooks the impact of real boundary conditions of the finite structure. Therefore, the STI method is a valuable tool for accurately quantifying the stop band behavior of real-world finite VAMM plates. This method takes into account the impact of real boundary conditions and finite length, disregarding the assumption of periodic boundary conditions.

## 10. Parameter Uncertainty Analysis

**Figure 17:**
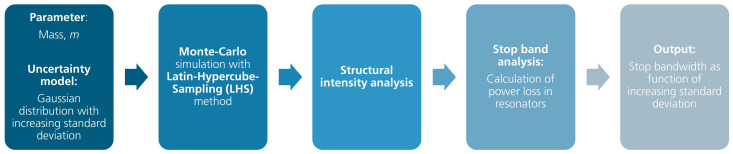
Simulation flow in uncertainty analysis.

The uncertainty quantification in this work is based on a one-factor-at-a-time (OAT) method, which means that only one parameter, uncertainty, is investigated. Here, the uncertainty in the resonator mass and its influence on the stopband width is of interest. Figure 17 provides an overview of the simulation flow using a power-based approach in uncertainty analysis.

In order to create uncertainties in the model parameter, a probability distribution function is required, from which the samples can be drawn. The probability density function of the uncertain parameter depends on the source of its uncertainty. Experimental characterization of uncertainty is needed to appropriately define the distribution function. In the absence of such detailed characterization, a normal or Gaussian distribution is considered to characterize the parameter uncertainty [52].

The uncertainty in the parameter is described by a normal distribution function and quantified by the mean, μ, of the distribution, which denotes the nominal value of the uncertain parameter, and its standard deviation, σ, which is proportional to the uncertainty of the parameter. The uncertainty level can be expressed by the coefficient of variation (CV), which is also known as the normalized standard deviation. The CV is defined as the ratio of the standard deviation to the mean, $CV=\frac{\sigma }{\mu }$ .

In order to accurately represent the output distributions, in this case, the stopband width corresponding to the uncertainty of the input parameter, the sampling technique, and the number of samples are crucial for the accuracy of the uncertainty analysis. Instead of using the random sampling method used in a classical Monte Carlo simulation, the Latin hypercube sampling (LHS) method is chosen, which is based on stratified sampling. Compared to pure random sampling, the LHS method eases the computational burden by sampling more effectively, thereby reducing the number of samples needed [52].

Based on LHS, masses for 25 resonators will be selected from the normal distribution of the mass described by its mean and standard deviation, N (μ, σ). In this study, the maximum CV value of 0.5 is chosen in order to evaluate the impact of larger uncertainty. During sampling, if the chosen mass has a value less than 0 g, the mass of the resonator is set to 0 g. The purpose of this very high maximum standard deviation is to find a general correlation between the variability of mass and the width of the stop band. It may not necessarily represent real manufacturing uncertainties.

During the Monte Carlo simulations, the CV is increased at a step size of 0.01, dividing the CV into 50 intervals $\left({CV}_{\max}/50\right)$. To achieve an accurate output probability distribution, it is necessary to have a considerably high number of samples for each CV value. Taking into consideration the computational burden of performing the STI analysis, 40 simulations are performed for each CV value. This means that the STI of the VAMM plate is computed 40 times for each CV before increasing the CV by 0.01. For each simulation, the probability distribution of the mass of the sample is calculated. The probability distribution follows a normal distribution and, therefore, has a slightly different mean and standard deviation compared to the distribution function from which these samples are drawn. The probability density function of the 25-resonator mass is of particular interest since it demonstrates the actual variation among them.

The power loss, $P_{\mathrm{loss},\mathrm{Rel}}$, is computed, and the stopband width is determined for the two threshold values, 90%, and 99%. The stopband widths for all simulations are plotted as functions of mass uncertainty, expressed as the CV of the input parameter, (σ/μ). In the case of an ideal damped finite VAMM plate where there is no variation in the mass of the resonators, a single stop band is present. However, with increasing uncertainty, there is the possibility that two or more stop bands will be identified. Since the influence of uncertainty is evaluated in terms of stopband width, the stopband widths of two or more stop bands are added to obtain the total stopband width. The larger deviation of the mass of the resonators affects the natural frequencies of the individual resonators and the mass ratio of the resonators to the host structure. This leads to the formation of more than one stop band.

**Figure 18:**
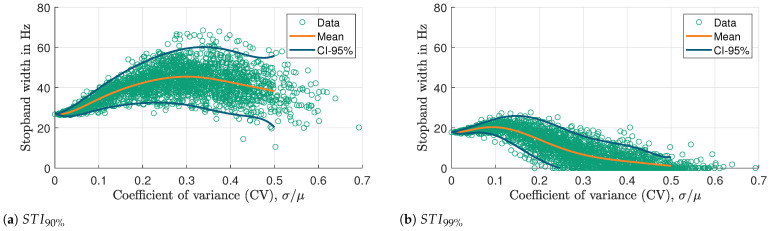
Stopband width as a function of CV for two different threshold values: (**a**) 90% and (**b**) 99%.

Figure 18 shows the stopband width as a function of CV for two different threshold values (a) 90% and (b) 99%. Inconsistencies in the mass of the resonators shift the resonance frequency of individual resonators, causing all resonators not to work on the same frequency anymore. This widens the stop band at the expense of the stop band depth. It is noticeable that the maximum CV for this simulation is above 0.5. Although, the maximum specified CV for the normal distribution is 0.5, the actual CV of each sample for the 25 resonators may differ from this value. The higher the specified CV, the greater the likelihood that the sample’s CV will deviate greatly from this value. The 95% confidence interval (CI) is indicated by the two blue lines, which signifies that 95% of the time, the stopband width will lie within this range. It is useful to examine the fluctuation of the mean stopband width values in relation to their corresponding standard deviations, in order to estimate the maximum achievable mean stopband width given an acceptable maximum variation in resonator masses.

In Figure 18a, the mean width of the stop band increases and reaches a peak at a CV value of 0.3 before decreasing again. However, the mean width always remains higher than the ideal stopband width (27 Hz). Similarly, in Figure 18b, the mean width of the stop band reaches a maximum CV value of 0.1 before decreasing below the ideal stopband width (18 Hz), eventually nearing zero. This indicates that the VAMM performance has significantly decreased, and it cannot achieve the desired stop band with 99% power loss. Moreover, the comparison of the confidence intervals in Figure 18a,b reveals that the CI 95% in Figure 18a is generally wider than that in Figure 18b.

By investigating the variation of mean stopband width values with respect to their corresponding standard deviations, the maximum allowable variation in resonator masses can be estimated while still achieving the required mean stopband width. This can be useful in the design and optimization of VAMM structures to achieve the desired degree of vibration attenuation, where the stopband width is an important performance parameter.

Based on the ${STI}_{99\%}$ method (Figure 18b), for the VAMM structure where great vibration reduction is the goal, the tolerances on the mass fabrication process must be tightened to ensure that the resonator masses are more consistent, thereby improving the reliability of the VAMM performance. On the other hand, the ${STI}_{90\%}$ method (Figure 18a) indicates that larger fabrication tolerances could be positively exploited to achieve a wider stop band range while still fulfilling the desired performance level at lower manufacturing costs.

## 11. Conclusions

This study presents a novel methodology for quantifying the power flow in a VAMM plate using the active STI integrated with FEM. The study was conducted on a rectangular Kirchhoff–Love plate model made of steel and excited by a point force. The resonators attached to the middle section of the plate were tuned to create a stop band in the frequency range of around 130 Hz. By utilizing the active STI, the relative power loss caused by the resonators in comparison to the host structure was calculated, predicting the stop band of the VAMM plate. Validation of the STI results with other approaches, such as the negative effective mass method, the UCDA method, and the frequency response function method for both undamped and damped systems, has shown that the STI method is a valuable tool that can be used to reliably predict the stop band of a finite VAMM structure. Even though this power-based approach is computationally intensive, it is a powerful and reliable tool used for predicting the stop band of real, complex 2D structures, considering damping, real boundary conditions, non-uniformities in the structure, and not depending on periodicity. This work also establishes the definition of a stop band in terms of percentage, which can be leveraged for a variety of applications based on the intended purpose and desired degree of vibration attenuation of the VAMM structure.

In the second part of this work, a robust algorithm based on relative power loss in VAMM was presented to evaluate and determine the impact of parameter uncertainties on the stop band behavior of a finite VAMM. Based on the $STI_{99\%}$ method, an increase in mass uncertainty up to a CV of 0.1 has a positive effect on the stopband width. However, if the mass uncertainty increases beyond this value, the performance of VAMM deteriorates significantly. On the other hand, the $STI_{90\%}$ method demonstrates that the variability in the mass uncertainty can be utilized in widening the width of the stop band, where the focus is on reducing vibrations by 90% compared to the host structure. In short, the relationship between the parameter uncertainties and the stopband width can be employed in the design and optimization process of VAMM, such as in determining the maximum allowable uncertainty while still achieving the desired level of vibration reduction. In order to study the impacts of other parameter uncertainties, such as spring stiffness, damping ratio, and the position of resonators on the stop band behavior, this proposed uncertainty quantification algorithm can be easily implemented. The findings can be useful in industrial applications for determining whether to loosen or tighten the manufacturing tolerances based on the sensitivity of the stopband width to parameter variations. In the future, the developed framework will be implemented on a more complex VAMM structure.

## Figures and Tables

**Table 1:** Summary of material parameters.

Property	Nomenclature	Value	Unit
Mass density	ρ	7850	kg/m^3^
Young’s modulus	E	204 × 10^9^	N/m^2^
Poisson’s ratio	ν	0.28	-
Damping ratio	η	0.01	-

**Table 2:** Comparison of UCDA for a single UC and five damped UCs.

	Single Unit Cell	Five Unit Cells
Schematic diagram	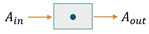	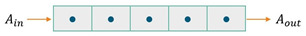
Amplitude reduction in %	$A_{\mathrm{red}}=e^{-\mu_{R}}\cdot 100$	$A_{\mathrm{red}}=e^{n\left(-\mu_{R}\right)}\cdot 100$

**Table 3:** Comparison of the applicability of different prediction methods for different conditions.

Conditions	$m_{\mathrm{eff}}$	${\mathrm{UCDA}}_{\mathrm{inv}}$	${\mathrm{UCDA}}_{\mathrm{dir}}$	STI
Undamped	✓	✓		
Damped			✓	✓
Periodicity	✓	✓	✓	✓
Non-periodicity				✓
Real Boundary Conditions				✓

**Table 4:** Comparison of the stop band prediction methods for undamped and damped systems.

Type of System	Stop Band Prediction Method	Stop Band Definition	Stop Band (Hz)	
			Lower Limit	Upper Limit
Undamped	$m_{\mathrm{eff}}$	$m_{\mathrm{eff}}<0$	130.0	143.0
	${UCDA}_{\mathrm{inv}}$	No solutions for ω	126.7	142.6
	${STI}_{99\%}$	$P_{\mathrm{loss},\mathrm{Rel}}>99\%$	126.4	142.1
	${STI}_{90\%}$	$P_{\mathrm{loss},\mathrm{Rel}}>90\%$	122.5	145.9
Damped	$UCDA_{\mathrm{dir}-99\%}$	$E_{\mathrm{red}}>99\%$	127.2	142.3
	${STI}_{99\%}$	$P_{\mathrm{loss},\mathrm{Rel}}>99\%$	125.3	143.2
	$UCDA_{\mathrm{dir}-90\%}$	$E_{\mathrm{red}}>90\%$	125.4	144.6
	${STI}_{90\%}$	$P_{\mathrm{loss},\mathrm{Rel}}>90\%$	120.7	147.4

## Data Availability

Data will be made available on request.
